# Characterization of genes required for both *Rpg1* and *rpg4-*mediated wheat stem rust resistance in barley

**DOI:** 10.1186/s12864-019-5858-z

**Published:** 2019-06-14

**Authors:** Shyam Solanki, Jonathan Richards, Gazala Ameen, Xue Wang, Atiya Khan, Harris Ali, Alex Stangel, Prabin Tamang, Thomas Gross, Patrick Gross, Thomas G. Fetch, Robert S. Brueggeman

**Affiliations:** 1 Department of Plant Pathology, North Dakota State University, Fargo, ND 58108-6050 USA; 2 Department of Plant Pathology and Crop Physiology, Louisiana State University AgCenter, Baton Rouge, LA 70803 USA; 3 Cereal Research Centre, Agriculture and Agri-Food Canada, 101 Route 100, Morden, MB R6M 1Y5 Canada

**Keywords:** Barley, Stem rust, Resistance, Exome capture, QTL, SKP1, F-box

## Abstract

**Background:**

*Puccinia graminis* f. sp. *tritici* (*Pgt*) race TTKSK and its lineage pose a threat to barley production world-wide justifying the extensive efforts to identify, clone, and characterize the *rpg4*-mediated resistance locus (RMRL), the only effective resistance to virulent *Pgt* races in the TTKSK lineage. The RMRL contains two nucleotide-binding domain and leucine-rich repeat (NLR) resistance genes, *Rpg5* and *HvRga1,* which are required for resistance. The two NLRs have head-to-head genome architecture with one NLR, *Rpg5*, containing an integrated C-terminal protein kinase domain, characteristic of an “integrated sensory domain” resistance mechanism. Fast neutron mutagenesis of line Q21861 was utilized in a forward genetics approach to identify genetic components that function in the RMRL or *Rpg1* resistance mechanisms, as Q21861 contains both genes. A mutant was identified that compromises both RMRL and *Rpg1*-mediated resistances and had stunted seedling roots, designated required for *P. graminis* resistance 9 (*rpr9*).

**Results:**

The *rpr9* mutant generated in the Q21861 background was crossed with the Swiss landrace Hv584, which carries RMRL but contains polymorphism across the genome compared to Q21861. To map *Rpr9*, a Hv584 x *rpr9* F_6:7_ recombinant inbred line (RIL) population was developed. The RIL population was phenotyped with *Pgt* race QCCJB. The Hv584 x *rpr9* RIL population was genotyped with the 9 k Illumina Infinium iSelect marker panel, producing 2701 polymorphic markers. A robust genetic map consisting of 563 noncosegregating markers was generated and used to map *Rpr9* to an ~ 3.4 cM region on barley chromosome 3H. The NimbleGen barley exome capture array was utilized to capture *rpr9* and wild type Q21861 exons, followed by Illumina sequencing. Comparative analysis, resulting in the identification of a 1.05 Mbp deletion at the chromosome 3H *rpr9* locus. The identified deletion contains ten high confidence annotated genes with the best *rpr9* candidates encoding a SKP1-like 9 protein and a F-box family protein.

**Conclusion:**

Genetic mapping and exome capture rapidly identified candidate gene/s that function in RMRL and *Rpg1* mediated resistance pathway/s. One or more of the identified candidate *rpr9* genes are essential in the only two known effective stem rust resistance mechanisms, present in domesticated barley.

**Electronic supplementary material:**

The online version of this article (10.1186/s12864-019-5858-z) contains supplementary material, which is available to authorized users.

## Background

The obligate biotrophic fungal pathogen, *Puccinia graminis,* causes the disease stem rust on a broad range of primary hosts including more than 365 species of cereals and grasses. While three *forma specialis* of *Puccinia graminis* cause disease on barley [1], wheat stem rust caused by *P. graminis* f. sp. *tritici* is considered one of the most serious disease of wheat and barley, because of historic epidemics that devastated crops, especially in the Upper Midwestern US in the early to mid-twentieth century. In the mid-twentieth century wheat breeders began pyramiding several resistance (R)-genes into varieties, providing durable resistance, which ended the occurrence of major epidemics on wheat. Stem rust was also managed by genetic resistance in barley, but only a single resistance gene, *Rpg1*, was deployed in the Midwestern US, protecting barley cultivars since 1942 [2].

The remarkably durable *Rpg1* gene was identified in 2002 by a positional cloning effort and was predicted to encode a cytoplasmic localized, constitutively expressed protein with two serine/threonine protein kinase domains, designated pK1 and pK2 [3]. The pK1 domain appeared to be a pseudokinase, lacking phosphorylation activity, whereas the pK2 domain was characterized as an active kinase. However, both domains were shown to function in stem rust resistance [4]. Interestingly, it was also shown that the RPG1 protein was systemically phosphorylated in vivo*,* five minutes post inoculation with avirulent stem rust urediniospores [5], but was not induced by virulent isolates. The RPG1 phosphorylation was required to elicit resistance response/s. Also, ubiquitin ligase mediated RPG1 protein degradation occurred between 20 and 24 h post inoculation (HPI) with avirulent isolates and was required to elicit the resistance response/s [6].

Although *Rpg1* maintained a remarkable level of durable resistance, for a single stem rust resistance gene deployed over vast cereal crop acreage, a new pathotype of *Pgt* designated race QCC (later given the five letter nomenclature QCCJB) virulent on barley containing *Rpg1* was identified in North Dakota in 1989 [7]. *Pgt* race QCCJB increased in prevalence and became one of the most common virulence types in North America causing disease epidemics [8]. The threat to barley production by this new stem rust race virulent on *Rpg1* prompted the evaluation of over 18,000 barley accessions from the USDA National Small Grains collection with the best source of *Pgt* race QCCJB resistance discovered in the unimproved barley line Q21861 from Queensland, Australia via CMMYT [9]. Genetic studies revealed that a single recessive gene, designated *rpg4*, conferred the resistance. The *rpg4* gene was also shown to be temperature sensitive providing resistance at lower (17–22 °C) temperatures [10], but was completely ineffective at temperatures above 27 °C. The *rpg4* gene was genetically mapped to the long arm of barley chromosome 5H [11].

A strain of stem rust, *Pgt* race TTKSK (also called as Ug99), emerged in Uganda, Africa in 1998 [12] that was considered highly virulent because it carried a unique combination of virulence genes enabling it to infect more than 80% of the wheat grown worldwide [13, 14] and more than 97% of barley cultivars, including those having *Rpg1* [15]. The threat posed by *Pgt* race TTKSK, mainly to wheat production, raised concerns for world food security [16]. Fortunately, for barley production, which is especially vulnerable to races virulent on commercial wheat varieties due to inoculum build up, the *rpg4*-mediated resistance locus (RMRL) in barley line Q21861 also confers effective resistance against *Pgt* race TTKSK [17] and its lineage.

Line Q21861 also contains resistance to isolates of rye stem rust*,* including *Pgs* isolate 92-MN-90, which was designated the *Rpg5* gene. The single dominant resistant gene was shown to be tightly linked to *rpg4* [18] and it was initially reported that *rpg4* and *Rpg5* could be the same gene despite their different inheritance, recessive vs dominant, respectively. The high-resolution recombinant lines used for positional cloning of *Rpg5* [19, 20] and *rpg4* behaved similarly to *Pgt* race QCCJB at the seedling stage and *Pgt* race TTKSK at both the seedling and adult plant stages suggesting that the same gene/s function in RMRL-mediated *Pgt* race QCCJB and TTKSK resistance.

Utilizing positional cloning, *Rpg5* was delimited to an ~ 70 kb region of the genome harboring three tightly linked genes including two nucleotide-binding domain and leucine-rich repeat (NLR) genes and an actin depolymerization factor. Allele analysis and post transcriptional gene silencing determined that the *Rpg5* rye stem rust resistance gene, one of the NLRs, contained an additional C-terminal integrated serine / threonine protein kinase (STPK) domain [21]. When *Rpg5* was originally identified, it was determined to be distinct from *rpg4* because high-resolution mapping based on 5232 gametes and RFLP markers showed *rpg4* segregating 0.11 cM away from *Rpg5* with an actin depolymerization factor, *HvAdf2*, reported as the best *rpg4* candidate gene [19, 22]. However, genomic sequence comparison at RMRL identified several SNP markers that when used to saturate the region showed that *HvAdf2* was not *rpg4.* The SNP marker saturation also determined that the *Rpg5* gene is required for *rpg4*-mediated wheat stem rust resistance [21] and is the functionally polymorphic *R*-gene at the RMRL [20].

The cloning and characterization of RMRL determined that the two resistances, *rpg4-*mediated wheat stem rust and *Rpg5*-mediated rye stem rust resistance mechanisms depend on *Rpg5* as the R-gene component [23] since its polymorphic nature in resistant and susceptible barley lines governs immune response. Another NLR gene *Rga1* in the RMRL locus is also required for QCCJB resistance yet didn’t contain resistance defining sequence polymorphism. Interestingly, the recent identification of the required for *rpg4-*mediated resistance 1 (*Rrr1)*, gene proximal to RMRL region was reported to be required for RMRL mediated resistance. *Rrr1* is also required for *Rpg1*-mediated resistance in the presence of the RMRL introgression [24]. These data suggest that the RMRL and *Rpg1* resistance mechanisms interact or have common signaling components. We hypothesize that the only two effective stem rust resistance genes characterized in barley to date, *Rpg1* and RMRL, confer broad spectrum resistance mediating early defense response mechanisms with spatial, temporal and functional hallmarks of non-host or slow rusting resistance mechanisms [25, 26]. Both resistances appear to be non-hypersensitive response (HR) resistance mechanism (Solanki et al., unpublished) indicative of a pathogen associated molecular pattern (PAMP) triggered immunity (PTI)-like resistance response. However, *Rpg1-*mediated resistance was previously suggested to be a (HR) dependent resistance mechanism more indicative of an effector triggered immunity (ETI) response [7], but our recent histology data suggests otherwise (Solanki et al., unpublished).

The plant innate immunity system has been separated into distinct layers with PTI acting as the first line of defense which has been classified as an active form of non-host resistance because of the recognition of conserved microbial molecules providing resistance to broad taxa of potential pathogens. This early defense mechanism is triggered at the cell surface by recognition of extracellular PAMP effectors, that induce responses including H_2_O_2_ accumulation, pathogen related (PR) gene expression, callose deposition at the point of ingress, and sometimes a low amplitude PCD response that is typically limited to a small number of cells surrounding the infection site [27]. These general PTI responses are activated by conserved transmembrane cell surface receptor complexes known to contain pattern recognition receptors (PRRs). The PRRs typically contain an extracellular receptor domain, a transmembrane domain and an intracellular kinase-signaling domain and are known as the receptor-like kinases (RLKs). Because, the broad *Rpg1*-mediated resistance responses are so rapid, within minutes of avirulent spores reaching the plant surface [6] it was hypothesized that there must be a cell surface receptor or receptor complex that recognizes the pathogen and relays the message to the cytoplasm activating Rpg1 phosphorylation and protein degradation. One of the main targets of the forward genetics approach employed in this study was to identify this putative cell surface receptor or members of the complex.

Host specific pathogens counter evolved virulence effectors to evade PTI responses by masking their PAMPs from detection, as observed for the chitin binding effector proteins [28, 29], or by blocking the signaling pathways as is seen with effectors that inhibit FLS2 PRR-mediated signaling following flg22 perception [30]. Fungal pathogens appear to secrete effectors into the host cell cytoplasm, or into the apoplast as is observed with the fungal chitin binding effectors, however, characterization of these mechanisms have yet to be elucidated at the functional level. Plants counter-evolved cytoplasmic localized immunity receptors, typically with NLR protein domain architecture, that recognize the presence of these effectors and elicit the hallmark higher amplitude PCD immunity response in plants, referred to as the hypersensitive response (HR) [31]. Once an effector is recognized by a cognate NLR immunity receptor, then it becomes an avirulence protein. For biotrophic pathogens that require living host cells to feed, these effectors that elicit programmed cell death (PCD) responses no longer facilitate disease, but hinder the pathogen by eliciting the plant immunity responses [32].

Fast neutron (FN) irradiation mutagenesis results in genomic DNA deletions from 1 bp (base pair) to several Megabase [33–35]. A FN induced mutant in the Q21861 background was identified with compromised RMRL specific *Pgt* race QCCJB resistance [36] and *Rpg1* specific *Pgt* race HKHJC resistance, designated as the required for *P. graminis* resistance 9 (*rpr9*) mutant. The *rpr9* mutant still retains a functional RMRL locus and *Rpg1* gene, thus, the mutant phenotype was due to a deletion within, or containing, an important gene required for RMRL and *Rpg1* function. This mutant also has a stunted root phenotype, short roots at early growth stages. Mapping of the mutation/s responsible for the *rpr9* and stunted root phenotypes in a Hv584 x *rpr9* RIL mapping population determined that the two mutant phenotypes co segregated to the same genetic interval. Thus, our identification of the *rpr9* mutant gene/s also identified candidate genes effecting the physiology of root development as well.

Here we report on the identification of ten candidate *rpr9* genes via genetic mapping and exome capture using a “mapping by sequencing” approach. To rapidly identify the deletion within or containing the *Rpr9* gene, the barley NimbleGen (Roche) exome capture array was utilized on the *rpr9* mutant and wildtype Q21861 and comparative sequence analysis identified a 1.052 Mbp deletion in the *rpr9* delimited region. Based on the barley POPSEQ positions of the genes in the deletion block and the intact genes flanking the deletion, a genetic to physical comparison identified ten high confidence genes classified into a family of four peroxidases, two NAD(P)H-quinone oxidoreductases, a SKP1-like 9 protein, a F-box family protein, a L-tyrosine decarboxylase and a RNAase protein. We propose that our top candidates encode the *SKP1-*like 9 and F-box proteins which were shown to work together as component of a SCF (SKP/Cullin/F-box and ring finger protein Rbx1) E3 ubiquitin ligase complex which have been shown to play roles in early plant immunity and root development pathways. RPG1 resistance relies on protein degradation after pathogen recognition, linking to the findings reported here, considering that our candidate mutant genes are components of the SCF E3 ubiquitin ligase complex and disrupt *Rpg1*-mediated resistance. The genetic analyses and tools developed here will facilitate the identification and functional validation of the *rpr9* gene and the gene/s responsible for the stunted root phenotype.

## Results

### Identification of mutants that compromise RMRL and *Rpg1* mediated resistance responses

**Fig. 1:**
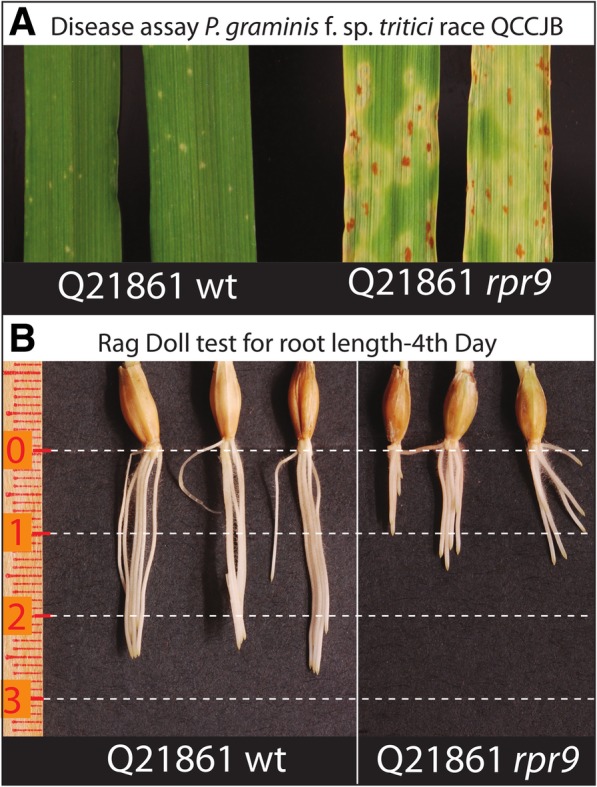
Stem rust seedling disease and root length phenotyping on wild type Q21861 and Q21861 *rpr9* mutant seedlings. **a** Seedling disease assay on the resistant barley line Q21861-wildtype and loss of RMRL resistance mutant Q21861-*rpr9*. **b** A representative picture of three germinated seeds and their root length determination by the Ragdoll test for wildtype Q21861 and the Q21861 *rpr9* mutant. The short root length phenotype was found to be associated with the *rpr9* mutant compared to wild type Q21861

The phenotyping of the Q21861 fast neutron (FN) irradiated population at the M_2_ generation with *Pgt* race QCCJB identified ten putative mutant individuals with compromised RMRL-mediated resistance that were designated as containing the required for *P. graminis* resistance 8–17 (*rpr8*-*rpr17*) genes. The original *rpr9* M_2_ mutant exhibited an average susceptible infection type (IT) of 3–2 (Fig. 1a) with *Pgt* race QCCJB. A *rpr9* M_3_ individual was backcrossed to wt Q21861 for four generations, which required phenotyping at each BCF_2_ generation with *Pgt* race QCCJB. The resulting BC_4_F_2_ *rpr9* mutant with the background mutations cleaned up exhibited ITs ranging from 2 to 3 to 3- with a mode of 3–2 compared to the highly resistant wild type line Q21861, which showed ITs ranging from 0; to 0;12 with a mode of 0; (Fig. 1a; Table 1). The line Q21861 is resistant to *Pgt* race QCCJB due to the RMRL and is also resistant to *Pgt* race HKHJC due to the *Rpg1* specific resistance [3]. Interestingly, *rpr9* also exhibited susceptible ITs to *Pgt* race HKHJC ranging from 2, 1 to 3- with a mode of 3-, 2, 1 compared to the resistant wild type line Q21861 which shows ITs ranging from 0;1 to 0;12 with a mode of;1 (Table 1). Thus, the *rpr9* mutant generated in the wheat stem rust resistant barley line Q21861 background, which carries both the *Rpg1* and RMRL resistance genes [3, 21, 22, 37], is compromised for resistance (Table 1) to both *Pgt* race QCCJB, specifically avirulent on RMRL [17] and HKHJC that is specifically avirulent on *Rpg1* [3, 19]. The Hv584/*rpr9* F_2:6_ RIL population was phenotyping with *Pgt* race QCCJB (Additional file 1: Table S1) and used for *rpr9* and the stunted root mutant QTL mapping *rpr9* qualitative gene mapping.

**Table 1:** Stem rust *Puccinia graminis* f. sp*. tritici* races QCCJB and HKHJC were used for disease phenotyping on wild type Q21861 and the *rpr9* mutant

Genotype	Rust race		Genotype	Rust race	
	*QCCJB*	*HKHJC*		*QCCJB*	*HKHJC*
*rpr9*	213-	3-,21	Q21861	0;1	0;1
*rpr9*	3-,2	3-,2	Q21861	0;1	2,1
*rpr9*	3-,2	33–2	Q21861	0;	;12
*rpr9*	2	23–1	Q21861	0;1	1;2
*rpr9*	23-	3-,2	Q21861	0;	;1
*rpr9*	2	2,1	Q21861	0;	0;1
*rpr9*	2	3-,2	Q21861	0;	;1
*rpr9*	2	3-,21	Q21861	0;1	1;
*rpr9*	2	3-,21	Q21861	0;12	1;
*rpr9*	3-,2		Q21861	0;12	;1
*rpr9*	3-,2		Q21861	0;1	0;1
			Q21861	0;1	
			Q21861	0;	

### Identification of stunted root growth phenotype associated with *rpr9*

**Fig. 2:**
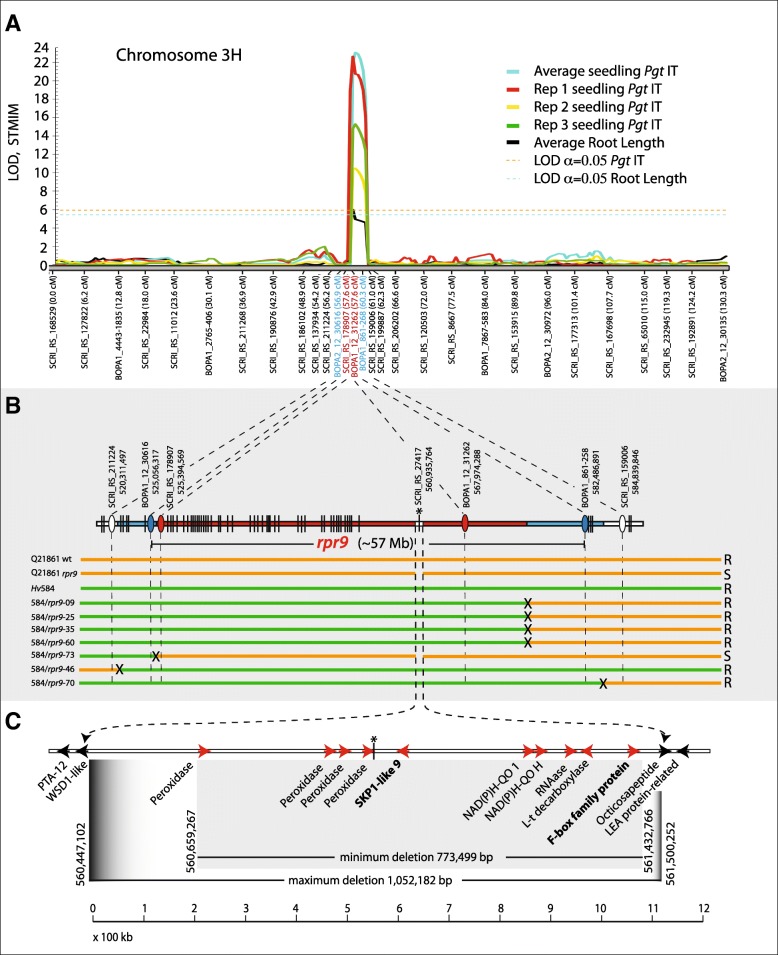
QTL and qualitative mapping of the *Rpr9* region showing the deletion identified within the region. **a** The Y-axis represents LOD values and X-axis the chromosome 3H map with markers designated below. Three replications and average of *Pgt* race QCCJB infection types with stunted root length QTL mapping are shown. The colors representing individual reps and averages are shown in the upper right. Blue indicate flanking and red indicate markers associated with the QTL. **b** The seven recombinants delimiting *rpr9* with Hv584/*rpr9* designations are listed on the left with the Q21861-*rpr9* and Hv584 parental lines and Q21861 wild type. The horizontal bars represent the genotypes of each (orange = Q21861 genotype and green = Hv584 genotype for markers listed above. The general reaction to *Pgt* race QCCJB is shown on the right (R = resistant and S = susceptible). Black Xs represent recombination regions. The horizontal bars above shows the cosegregating blocks of markers with the most distal and proximal *rpr9* cosegregating markers based on genome sequence positions denoted with a red ovals and other markers shown as vertical black lines. Blue ovals denote flanking markers with the smallest physical interval. White ovals denote the next segregation blocks proximally and distally. All markers are shown in their approximate physical positions. The asterisk denotes the SCRI_RS_27,417 marker that is presents within the *rpr9* deletion. **c** The deletion covering ten high confidence annotated genes, with functions listed below and the top *rpr9* candidates indicated in bold lettering. The horizontal white bar represents the genome sequence of cv Morex with red arrows representing the relative positions and directionality of annotated *rpr9* candidate genes. Black arrows the flanking genes that were present in both wildtype Q21861 and the *rpr9* mutant delimiting the deletion. The black lines below denote the minimum (0.773 Mbp) and maximum (1.052 Mbp) deletion size. The 100 kilobase scale is shown below

Germination of *rpr9* mutant seed on petri plates lead to the observation that they exhibited a stunted root growth compared to wildtype Q21861 seed (Fig. 1b). After four rounds of backcrossing the mutant to wildtype Q21861 and selection for susceptibility to *Pgt* race QCCJB the stunted root phenotype was still present suggesting that it could be determined by the same mutation that compromised RMRL- and *Rpg1*-mediated resistance. The calculated mean root length of fourteen *rpr9* mutants was 16.5 mm, whereas wt Q21861 had a mean root length of 25.64 mm. The difference in root length was found to be statistically significant (two tailed *P* < 0.05) between *rpr9* and wt Q21861 using an unpaired *t*-test (Table 2). Further, root length was recorded for three seeds of each of the 81 Hv584/*rpr9* RIL individuals, the parental lines *rpr9* and Hv584 and wildtype Q21861. The average root length data from germinated seeds were used for QTL mapping (Fig. 2; Additional file 1: Table S1).

**Table 2:** Statistical determination of difference in the root length in Q21861 and *rpr9*

Group	*rpr9*	Q21861
N (Sample size)	14	14
Mean root length	16.5	25.64
SD	1.65	1.82
SEM	0.44	.49

An unpaired *t-*test was used to determine if the difference of root length (in mm) at the 4th day of germination was significantly different between the *rpr9* mutant and wildtype Q21861. The difference between the root lengths were statistically significant (two tailed *P* value = 0.0065). SD- Standard deviation, SEM - Standard error of mean

### Linkage mapping and QTL analysis for *rpr9* and stunted root phenotype

To determine if the *rpr9* phenotype was contributed by a single recessive mutation, the Swiss landrace line Hv584 that contains a functional RMRL [38] was crossed with the *rpr9* mutant. Fifty-four Hv584/*rpr9* F_2_ individuals independent from the F_2_ individuals advanced for the RIL population development were phenotyped with *Pgt* race QCCJB showing a 12 resistant: 42 susceptible ratio which fit the expected 1:3 ratio (χ^2^ = 0.637) for a single recessive *rpr9* mutation. The recessive phenotype was observed upon fast neutron mutagenesis and deletion of the *Rpr9* gene. The mapping of the *rpr9* stem rust susceptibility and stunted root growth phenotype was performed utilizing the barley iSelect 9 k Infinium chip genotyping data of the Hv584/*rpr9* F_6_ RIL population made up of 81 individuals. A robust genetic linkage map was generated with 2701 polymorphic markers distributed across the seven-barley chromosome representing ~ 30% polymorphism for the 9000 iSelect markers on the 9 k Infinium chip (Additional file 2: Table S2).

Following removal of co-segregating markers, the final linkage map used for QTL analysis consisted of 563 non-redundant loci spread across all seven barley chromosomes (Additional file 3: Figure S1). QTL analysis identified an ~ 3.4 cM region harboring the *Rpr9* gene as well as the mutation resulting in the stunted root phenotype. We identified a significant QTL (LOD = 24,α_0.05_ = 6) on barley chromosome 3H for seedling *Pgt* race QCCJB resistance (Additional file 4: Figure S2) delimited by the co-segregating block represented by non-redundant flanking markers BOPA1_12_30616 (12_30616) and SCRI_RS_159006 (Fig. 2a) covering POPSEQ positions from 59.63 cM to 75.92 cM representing a physical region of ~ 59.78 Mbp from positions 525,056,438 -584,839,846 bp on chromosome 3H. This QTL harbors two SNP markers SCRI_RS_178907 (LOD = 21.99) and BOPA1_861–268 (LOD = 18) representing two different co-segregating blocks of 49 and 4 markers respectively (Additional file 2: Table S2) underlying the *rpr9* region. For the fine mapping using *rpr9* phenotyping as a qualitative marker seven critical recombinants (Hv584/*rpr9*–09, Hv584/*rpr9*–60, Hv584/*rpr9*–35, Hv584/*rpr9*–25, Hv584/*rpr9*–46, Hv584/*rpr9*–73 and Hv584/*rpr9*–70) were identified, further delimit it to the co-segregating block of 49 markers represented by SCRI_RS_178907/ BOPA1_12_31262 on the QTL map (Fig. 2a and b). The most proximal marker within the *rpr9* cosegragating markers based on the genome assembly was SCRI_RS_178907 (POPSEQ position ~ 59 cM, physical start position on chr. 3H at 525,394,569 bp) and most distal markers was BOPA2_12_31262 (POPSEQ position ~ 68 cM, end position on barley physical map is chr. 3H at 567,974,409 bp) representing a physical region of ~ 57.43 Mbp on chr. 3H (Fig. 2b). This co-segregating block was flanked by the markers BOPA2_12_30616 (525,056,438 bp) 0.7 cM proximal of *rpr9* and BOPA1_861_268 (582,486,654 bp) 0.7 cM distal of *rpr9* delimiting the region to an ~ 57.44 Mbp region (Fig. 2b).

Interestingly, the only significant seedling short root QTL (LOD = 6, α_0.05_ = 5.74) was also flanked by the markers BOPA1_12_30616 (LOD = 4.4) and BOPA1_861–268 (LOD = 4.5) on chromosome 3H (Fig. 2a, Additional file 5: Figure S3). Thus, from here forth we will refer to the *rpr9* locus as governing, RMRL and *Rpg1*-mediated resistance as well as the stunted root phenotype.

### Identification of candidate deleted genes using exome capture

As multiple wt and mutant genotypes are barcoded and sequenced in parallel on a single Illumina Nextseq flow cell, of the ~ 700 million reads generated, a total of 115,741,280 sequencing reads were identified as wildtype Q21861 and 104,846,733 reads were from the *rpr9* mutant, representing a very balanced exome capture sequencing library. After QC and trimming the sequences averaged 151 bases per read. Alignment of the reads to the Morex draft genome sequence resulted in 88.79% of the wt Q21861 and 82.60% of the *rpr9* reads aligning to the reference barley genome.

**Fig. 3:**
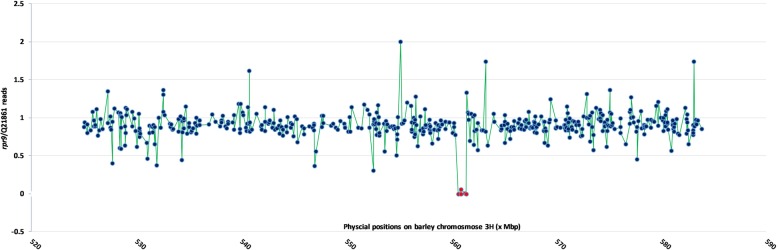
A graphical representation of ratio of exome capture read in *rpr9* mutant and Q21861 wildtype. The X axis represents the physical sequence position (x Mbp) on barley chromosome 3H in the *rpr9* region, whereas Y axis represents the ratio of total exome capture coverage reads of 487 high confidence annotated genes between *rpr9* mutant and Q21861-wt. Blue circles represents each gene in the region. Red circles represent the deleted genes in the *rpr9* region with a very low coverage ratio (< 0.05)

The pipeline that calculates captured unique sequence coverage across all exome capture targets (Additional file 6: Table S3) identified a large block of deleted genes within the *rpr9* region delimited by the genetic map (Fig. 3).

The deletion spanned a physical region of 1.052 Mbp based on the positions of two genes, HORVU3Hr1G074910 (end position on the barley physical map ch. 3H - 560,448,070) and HORVU3Hr1G075060 (start position on the barley physical map ch. 3H - 561,500,252) that flank the deletion yet are still present in the *rpr9* mutant (Fig. 2c) based on the coverage ratio (cutoff ratio 0.05) of *rpr9* and Q21861 genes present in the QTL region (Additional file 6: Table S3). The region present between the most proximal and distal deleted genes, HORVU3Hr1G074920 (start position on the barley physical map ch. 3H – 560,659,267) and HORVU3Hr1G075050 (end position on the barley physical map ch. 3H – 561,438,786), respectively, on the barley physical map [39], show a minimum deletion of ~ 0.773 Mbp (Fig. 2c). Within this ~ 0.773 Mb deletion, eleven HC genes are annotated in barley reference genome. This block of eleven genes includes a predicted leucine-rich repeat receptor-like protein kinase (gene model HORVU3Hr1G074930) yet sequencing reads aligned to this gene model. The IPK barley high confidence gene list released in 2016 is utilized for exome data analysis and this receptor like kinase gene is annotated twice on chromosome 3H as HORVU3Hr1G074930 (560,737,798-560,739,850) and HORVU3Hr1G067050 (509,350,259-509,352,312). Upon thorough inspection we concluded that gene model HORVU3Hr1G074930 was identical to HORVU3Hr1G067050 and appears to be a mis-annotated gene in the *rpr9* deletion region on chromosome 3H. Thus, the deletion contained ten HC deleted genes (Table 3) including a SKP1-like 9 protein encoding, a NAD(P)H-quinone oxidoreductase subunit H, a NAD(P)H-quinone oxidoreductase subunit 1, a RNAase encoding, one L-tryrosine decarboxylase, an F-box family protein encoding gene and four peroxidase superfamily genes. Interestingly in our 9 k iSelect co-segregating marker panel for Hv584/*rpr9*, we identified a locus SCRI_RS_27,417 starting at the ch. 3H physical position 560,935,764 bp on the *rpr9* containing co-segregation block (de novo genetic position 57.55 cM) (Additional file 2: Table S2). This marker is situated in the identified *rpr9* deletion region according to the barley genome sequence. We found the physical position of marker SCRI_RS_27,417 in the 3′ untranslated region (UTR) of annotated mRNA HORVU3Hr1G074960.2 (560934358–560,935,865, chr. 3H) encoding for a peroxidase superfamily protein, present in the mapped *rpr9* locus. In our deletion analysis this gene had 4835 exome capture reads in wild type Q21861 across the HORVU3Hr1G074960 and 269 exome capture reads in *rpr9* mutants, thus giving a coverage ratio of 0.05 between *rpr9* and Q21861. However, in Q21861, coverage was across the entire coding determining sequence (CDS), while in *rpr9*, the 269 exome capture reads specifically aligned to the 3′ terminus of the gene with a very specific terminus at position 988 in the CDS. This read alignment suggested that the gene was at one end of the deletion suggesting that the gene ordering in the region may not be correct based on the genome sequence (Additional file 7: Figure S4).

**Table 3:** List of deleted and flanking genes present in *rpr9* deletion region on barley chromosome 3H

Gene Name	Start	End	Annotation	Exome capture reads		Ratio
				Q21861	*rpr9*	(*rpr9*/Q21861)
HORVU3Hr1G074850	560,316,839	560,326,306	plastid transcriptionally active 12	2574	2264	0.879
HORVU3Hr1G074910	560,447,102	560,448,070	O-acyltransferase (WSD1-like) family protein	13	10	0.769
HORVU3Hr1G074920	560,659,267	560,660,724	Peroxidase superfamily protein	4075	3	0.001
^a^HORVU3Hr1G074930	560,737,798	560,739,850	Leucine-rich repeat receptor-like protein kinase (LRK) family protein	0	0	0
HORVU3Hr1G074940	560,884,155	560,885,580	Peroxidase superfamily protein	4075	7	0.001
HORVU3Hr1G074950	560,902,589	560,904,079	Peroxidase superfamily protein	5063	5	0.001
HORVU3Hr1G074960	560,934,358	560,935,865	Peroxidase superfamily protein	4835	269	0.056
HORVU3Hr1G074970	561,006,112	561,007,794	SKP1-like 9	8	0	0
HORVU3Hr1G075000	561,254,447	561,255,632	NAD(P)H-quinone oxidoreductase subunit H, chloroplastic	0	0	0
HORVU3Hr1G075010	561,255,634	561,256,481	NAD(P)H-quinone oxidoreductase subunit 1, chloroplastic	0	0	0
HORVU3Hr1G075030	561,299,425	561,299,615	RNAase	0	0	0
HORVU3Hr1G075040	561,302,733	561,304,797	L-tyrosine decarboxylase	1588	12	0.007
HORVU3Hr1G075050	561,432,288	561,438,786	F-box family protein	945	2	0.002
HORVU3Hr1G075060	561,500,252	561,501,311	Octicosapeptide/Phox/Bem1p family protein	70	93	1.32
HORVU3Hr1G075070	561,614,572	561,615,955	LEA protein-related	361	384	1.06

List of eleven annotated high confidence genes present in the *rpr9* deletion region on barley chromosome 3H along with flanking genes in BARLEX database. The table contains the gene name, annotated physical sequence position for start and end positions for the coding *d*etermining sequence (CDS) on barley chromosome 3H, exome capture reads for Q21861 and the *rpr9* mutant with their ratio

^a^denotes the mis-annotated LRK gene present in the deletion region under the *rpr9* QTL

## Discussion

In wheat, nearly 60 stem rust resistance genes have been identified and hundreds of different races have been typed using single R-gene differentials. However, in barley only five stem rust resistance genes have been identified and of these only two, *Rpg1* and the *rpg4*/*Rpg5* complex or RMRL*,* have been shown to be effective. However, recent association mapping using landraces and wild barley populations [40] have identified some novel stem rust resistances. Although, *Rpg1* and RMRL confer effective resistances they still do not resemble the strong race specific resistances identified in wheat. The *Rpg1* and RMRL genes/loci confer broad-spectrum resistances, which are more similar to incomplete or slow rusting gene action and when combined currently provide resistance to all known races of wheat stem rust. We speculate that barley is a recent non-host to wheat stem rust and has not undergone a prolonged evolutionary molecular arms race with the adapted pathogen. This relatively short co-evolutionary history between barley and wheat stem rust has resulted in limited resistance sources in barley compared to the large number of race specific *R*-genes and multiple pathogen races correlating to interactions with specific resistances in wheat. The *Rpg1-* and RMRL-mediated resistance mechanisms may be forms of non-host resistance that do not fall into the usual class of typical race specific NLR rust R-genes. These race specific NLR resistances are typically post-haustorial resistance mechanisms that elicit strong ETI mechanisms associated with strong HR responses as characterized in the classic flax-flax rust model system [41] and the majority of stem rust resistance genes characterized in wheat.

In barley, the RMRL provides resistance against a broad spectrum of stem rust races including the highly virulent race TTKSK. To identify conserved genes that function in RMRL or *Rpg1* resistance, FN irradiation of barley line Q21861 (RMRL*+* and *Rpg1+*) was used to induce the deletion mutant *rpr9* that is susceptible to *Pgt* race QCCJB which is specifically avirulent on RMRL and is also susceptible to *Pgt* race HKHJC which is specifically avirulent on *Rpg1* containing barley lines. The *rpr9* mutant was utilized for genetic mapping via a Hv584 x *rpr9* cross followed by exome capture and mapping-by-sequencing to rapidly identify candidate *Rpr9* genes that underlie the mutant phenotypes. A combine approach of QTL and genetic mapping was used to identify the *rpr9* region of the barley genome. QTL mapping using the 9 k iSelect marker panel delimited *rpr9* to a ~ 59.78 Mbp region on chromosome 3H between the SNP markers BOPA2_12_30616 and SCRI_RS_159006 located at physical map positions from 525,056,438 -584,839,846 bp respectively. For fine mapping of the *rpr9* QTL we identified seven critical recombinants enabled to us to further resolve the position of *rpr9* on a co-segregating block of 49 loci covering an ~ 57 Mbp physical region. We also determined that the *rpr9* mutant exhibited a stunted root phenotype, as after cleaning the genetic background of *rpr9* with four rounds of backcrossing, both the stem rust susceptibility and stunted root phenotype were retained suggesting that the mutation-giving rise to both mutant phenotypes may not be distinct.

Root length is controlled by interaction between many cellular factors [42], thus identification of the only significant QTL for the stunted root phenotype in the Hv584/*rpr9* RIL population at the *rpr9* deletion region flanked by the two segregation blocks represented by SNP markers BOPA2_12_30616 and BOPA1_861–268 located at physical map positions from 525,056,438 -582,486,654 bp respectively, indicates that the same mutation event is pleiotropic and compromise both, the RMRL and *Rpg1* mediated resistance responses as well as the seedling root length growth. The distribution of root length in the RIL population was found to have a normal distribution rather than following a bimodal distribution (Additional file 8: Figure S5) suggesting that although the only significant root length QTL mapped to the *rpr9* deletion-locus causing shorter root length, the natural polymorphism present between Q21861 and Hv584 contributes to the segregating and continuous distribution of the root lengths within the RIL population. However, none of the natural polymorphism between Q21861 and Hv584 had a significant effect that was detected in the population, suggesting that our artificially induced polymorphism, using fast neutron mutagenesis, provided a greater phenotypic effect than any of the natural variation contributing to this complexly inherited trait.

Using the recently developed barley exome capture array followed by Illumina sequencing of the *rpr9* mutant compared with the wt Q21861, a deletion with a maximum length of ~ 1.05 Mbp was identified on chromosome 3H between the physical position of 560,448,070–561,500,252 bp underlying the *rpr9* delimited region. The deleted region contains ten high confidence genes along with a mis-annotated LRK. The ten high confidence genes were classified as a block of four peroxidases, two NAD(P)H-quinone oxidoreductases and one each of SKP1- like 9, a F-box family protein, a L-tyrosine decarboxylase, and a RNase family protein (Fig. 2c, Table 3). Interestingly in the 9 k iSelect co-segregating marker panel for Hv584/*rpr9*, we identified the SNP marker SCRI_RS_27,417 that was present in the identified *rpr9* deleted region, thus warranting further examination of the annotated deletion. Our analysis identified the position of the SNP marker in the 3′ UTR of annotated gene HORVU3Hr1G074960 (560934358–560,935,865, chr. 3H, CDS- 1074 bp). Since exome capture reads covered the entire CDS in wildtype Q21861 and abruptly started at position 988 in the last exon extending into the 3’UTR it appears that this gene is actually located at the terminal end of the deletion. This suggests that although the gene content of the region is correct the ordering of the genes at the region are incorrect. We have observed such discrepancies in the Morex draft genome assembly when fine mapping and sequencing of the *rcs5* spot blotch resistance locus [43] and RMRL (Solanki et al., Unpublished).

The genetic mapping and exome capture data shows that the stunted root phenotype and *rpr9* disease susceptibility phenotype are either governed by two different genes present in the deletion block or a single gene within the deletion is responsible for both mutant phenotypes. If a single gene controls both phenotypes, then it represents an interesting example of a pleiotropic effect. However, there are many examples of pleiotropic genes effecting both abiotic and biotic resistances as well as developmental processes [44, 45]. It has been hypothesized that since the *rpr9* mutant compromises both the very early *Rpg1*-mediated stem rust resistance mechanisms [5] as well as the early responses induced by RMRL there may be a cell surface receptor responsible for the early perception of the pathogen resulting in early pathogen perception at the leaf surface and rapid intracellular responses within minutes of the pathogen contacting the leaf surface. Therefore, upon our initial characterization of the deletion region the receptor-like kinase gene present in the region was of great interest and our initial top candidate gene. However, in the recently released IPK barley high confidence gene list utilized for exome data analysis, this receptor like kinase gene is annotated twice on chromosome 3H as HORVU3Hr1G074930.1 (560,737,798-560,739,850) and HORVU3Hr1G067050.1 (509,350,259-509,352,312) and both gene annotations are identical. We concluded that it is a mis-annotation at the 560,737,798-560,739,850 (HORVU3Hr1G074930.1) position present inside the *rpr9* region, further indicating the need to refine the barley whole genome assembly and annotation. Thus, we focused on the other candidate genes within the deleted region. Interestingly, two of the candidate genes are predicted to encode a *SKP1* (S-phase kinase- associated protein) and F-box proteins, known to be part of functional components of the multiprotein E3 ubiquitin ligase complex known as SCF (SKP/Cullin/F-box and ring finger protein Rbx1). SCF type E3 ubiquitin ligases are well characterized [46] and in SCF complexes the Cullin and Rbx1 proteins constitutes a scaffold core which is connected to the F-box proteins through SKP1 [47]. In the SCF complex F box proteins determine the specificity for ubiquitination thus are considered substrate recognition components [48]. In *Arabidopsis* 21 *SKP* genes (ASK) were predicted [49], however in yeast and human only one functional *SKP1* gene is present [50]. We found that in barley sixteen high confidence SKP genes were predicted upon a genome search (Additional file 9: Table S4).

Many studies suggested the role of SCF complex in regulation of plant immunity. Recent evidence of E3 ubiquitin ligase MUSE1 and MUSE2 controlling the dual NLR defense responses mediated by SNC1-SIKIC2 NLR pair indicate the importance of ubiquitination mediated regulation to regulate the fine switch between partner NLRs [51]. In barley *Rpg5* and *Rga1* NLR pair may have similar regulation mediated by SCF complex for *Pgt* resistance. In tobacco it has been reported that *NbSGT1* (*Nicotiana benthamiana* suppressor of G2 allele of SKP1) a highly conserved co-chaperone component of the SCF complex, interacts directly with *NbSKP1* (a component of the SCF ubiquitin ligase complex) and NbRar1 [52, 53]. *Rar1* is required for TNL (Toll/interlukin 1 receptor-Nucleotide binding-Leucine rich repeats) *N* gene mediated resistance in tobacco and also has been shown to function downstream of powdery mildew recognition and upstream of plant hypersensitive cell death responses upon H_2_O_2_ accumulation via several CNLs (CC-NB-LRR) including *Mla* mediated resistance in barley [54]. TNL and CNL proteins are involved in pathogen recognition and defense mechanisms yet have diverse signaling pathways. TNLs are rare in monocots and absent in cereals whereas CNLs are found in dicots. Thus, *Rar1* represents a signaling factor functioning in common pathways evoked by TNL and CNL resistance genes. In tobacco upon *SKP1* or *SGT1* silencing, *N* gene mediated resistance has been shown to be compromised for TMV resistance. *Triticum aestivum SKP1* (*TSK1*) was found to be expressed in young root and spikes and a low-level expression was detected in leaves. *TSK1* overexpression in *Arabidopsis* was shown to increase abscisic acid (ABA) responsive phenotypes such as stomatal closure, root growth and seed germination and enhanced drought tolerance [55], thus possibly functioning as a positive regulator of ABA signaling .

**Fig. 4:**
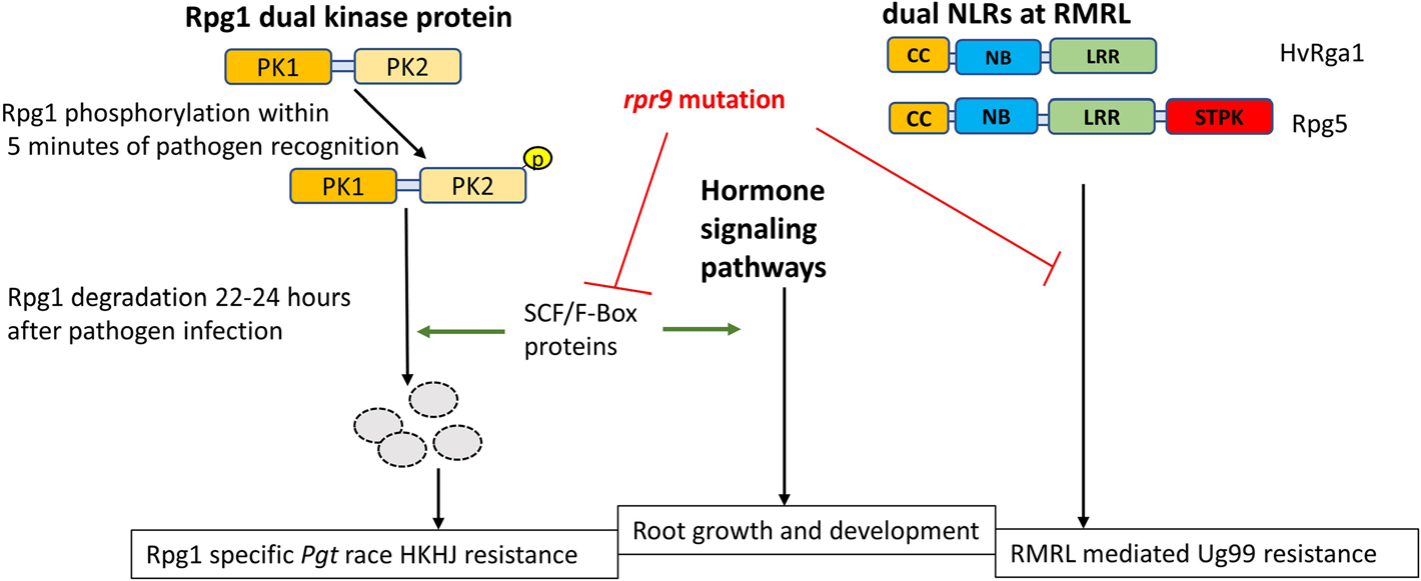
A hypothetical model of candidate rpr9 gene disrupting the RMRL, *Rpg1* and root length signaling pathway. Our topmost candidate *rpr9* genes are SCF and F-box protein coding, associated with ubiquitin mediated protein degradation pathway. Rapid phosphorylation of Rpg1 dual kinase protein followed by protein degradation is required for *Pgt* race HKHJ resistance. Similarly, Ubiquitination plays an important role in root development and hormone signaling. Thus, we hypothesize *rpr9* mutation disrupts the Rpg1, RMRL and hormone signaling pathway by inhibiting the possible intermediate ubiquitination step resulting in compromised phenotype

In barley five high confidence (HORVU3Hr1G075050.2, HORVU1Hr1G077600.2, HORVU4Hr1G052070.1, HORVU0Hr1G030450.1, HORVU1Hr1G068570.1) and one low confidence (HORVU3Hr1G079580.6) *F-box* genes were mined from the IPK database. In *Arabidopsis* 694 *F-BOX* genes were predicted [48] making it one of the largest protein families. F-box proteins show high functional diversity and are involve in many functions including pathogen perception and circadian rhythm. *Arabidopsis* DOR (DrOught tolerance Repressor) is a SFL (S-locus F box like) family protein and is expressed in the stomatal guard cells and shown to control the ABA biosynthetic pathway and negatively controls ABA induced stomatal closure under drought stress [56]. In another study the *Arabidopsis F-box-Nictaba* was shown to be a pathogen inducible gene and overexpression in plants showed resulted in reduced infection by *Pseudomonas syringae* pv. *tomato* strain DC3000 [57]. It was also shown that the F-box protein ACIF1 (Avr9/Cf-9–Induced F-Box 1) silencing suppressed the hypersensitive response triggered by a diversity of pathogen elicitors such as Avr9, AvrPto and the P50 helicase of TMV [58]. Thus, the E3 ubiquitin ligase SCF complex components identified within the deletion in the *rpr9* mutant region are certainly strong *rpr9* candidate genes possibly functioning downstream in the cellular pathways governed by the CNL Rpg5 and dual kinase protein Rpg1. It has been determined that *Rpg1*-mediated resistance is dependent upon RPG1 ubiquitination and subsequent E3-mediated degradation [6], thus the deleted SCF complex proteins may be responsible for the E3 ubiquitination required for *Rpg1* degradation post interaction with avirulent *Pgt* isolates. The Rpg1 protein degradation 24 h post infection is required for the resistant reaction to occur, thus, if these E3 ubiquitin ligase SCF complex components are *Rpr9*, then loss of Rpg1 degradation could be expected in the *rpr9* mutant post infection with *Pgt* race HKHJ if this hypothesis is correct (Fig. 4).

Although the SCF complex proteins are considered our best candidates there are other deleted genes in the region that are associated with biotic stress responses. Two other candidates are the NQO (NAD(P)H-quinone oxidoreductase) subunit H and subunit 1 that were previously show to be involved in electron transport in photosystem II and quinone detoxification [59, 60]. Also, the *Arabidopsis* L-tyrosine decarboxylase (TyrDC) was shown to be induced by wounding, drought stress and fungal effector perception [61–63] and is known as the first enzyme in the benzylisoquinoline alkaloids pathway. These metabolites act as antimicrobial compounds and cell wall reinforcement agents to provide immune function [64]. Peroxidases are well characterized in plant defense responses and have been shown to be involved in removal of hydrogen peroxide at the cell wall and initiate the wounding defense responses [27, 65–67]. Thus, future functional studies are warranted to validate the *rpr9* gene among the list of the ten candidate genes identified. Although *Rpg1* and RMRL confer some differential specificity [15, 68], the *rpr9* mutant suggests the two mechanisms contain common resistance component/s probably downstream in a converging resistance pathway.

## Conclusions

Virulent races of *Pgt* emerging in Africa, Asia, Europe including recent reports of *Pgt* in the United Kingdom after nearly 60 years, on barberry plants adjacent to barley fields, underlines the reoccurrence of this dangerous foe. In the present research we have successfully utilized the combination of fast neutron mutagenesis, genetic mapping and exome capture sequencing to rapidly identify conserved genes involve in RMRL- and *Rpg1*-mediated resistance responses, the only two effective *Pgt* resistances in barley which provide protection against a broad spectrum of *Pgt* races. We mapped and identified candidate *rpr9* genes, which is not only required for both RMRL*-* and *Rpg1-*mediated *Pgt* resistance but also confer a stunted root phenotype. Further validation of the candidate genes will provide insight into the functional mechanisms controlling both phenotypes possibly by one or distinct genes underlying *rpr9* mutation. The identification of genes involved in the resistance signaling mechanisms will fill knowledge gaps in downstream signaling mechanism in NLR pathways.

## Methods

### Mutant screens

The barley line Q21861 (PI584766) was utilized for the forward genetics screen because it carries the RMRL as well as the *Rpg1* stem rust resistance gene. After three generations of single seed descent and seed increase the genetic quality Q21861 seed (~ 3 kg) was irradiated with fast neutrons (3.5 or 4.0 Gy using protocol 563) at the FAO/IAEA Seibersdof SNIF facility near Vienna, Austria. Q21861 *M*_1_ seeds were planted and allowed to self and ~ 6000 spikes containing *M*_2_ seed were harvested from individual plants. In the greenhouse, 20–30 *M*_2_ seed from individual spikes were planted in cones filled with a peat moss:perlite (3,1 v/v) potting mix (#1 Sunshine Mix, Fisons, Vancouver, Canada). Plants were inoculated with pathotype *Pgt*-QCCJB when the first leaves were fully expanded and assessed for their infection types 12–14 days post inoculation according to Steffenson et al. [69]. Infection type (IT) were based on a 0–4 Stakman IT (Infection Type) scale used for barley [17, 70]. Where 0 is highly resistant and 4 is highly susceptible with the middle range numbers representing intermediate reactions, which are further modified by + or – and a fleck (;) indicating a small necrotic area. IT1 indicates minute uredinia; IT2 small uredinia with chlorosis; IT3 medium uredinia often with chlorosis; and IT4 indicates large uredinia with chlorosis. Barley often exhibits mesothetic reactions with two or more ITs on a single leaf therefore ITs observed are recorded in order of their prevalence and this categorical disease rating was converted to a single numerical value as described previously [38]. When segregation for stem rust reactions were observed within an individual *M*_2_ spike, single heads were harvested and phenotyped with pathotype *Pgt*-QCCJB at the *M*_3_ generation. A consistent mutant was identified and designated as *rpr9*.

### Segregation analysis and RIL population development

The *rpr9* mutant generated in the Q21861 background was backcrossed to Q21861 for four generations and fifteen BCF_2_ individuals were phenotyped at each back cross (BC) generation to identify homozygous *rpr9* mutant individuals. Pollen from the *rpr9* mutant with the background mutations cleaned up with four generations of backcrossing was crossed with the Swiss landrace Hv584 (UMN Pathology breeding program) [70] as the female parent to generate the Hv584/*rpr9* population. The Swiss landrace *Hv*584, is resistant to *Pgt* races QCCJB and TTKSK because it carries RMRL [70] but contains polymorphism across the genome compared to Q21861. Seeds of all the barley genotypes used in the study are available at Barley Pathology, NDSU. For inheritance studies 54 Hv584/*rpr9* F_2_ individuals were phenotyped at the seedling stage with *Pgt* race QCCJ in the growth chamber as described in Mirlohi et al., (2008) [37]. The single gene segregation analysis was calculated utilizing a χ^2^ test with the null hypothesis that the *rpr9* phenotype was contributed by a single recessive gene. A Hv584/*rpr9* RIL population consisting of 95 individuals was developed through single seed decent till the F_5:6_ generation and phenotyped for QCCJ seedling disease assay.

### Rag-doll test for root length analysis

Two seed germination papers/brown paper towels were placed together, and a horizontal line was drawn using a pencil at the center of the paper. The paper towels were moistened with water. Twenty *rpr9* and wt Q21861 seed were selected randomly and placed separately on one half of the moist germination papers keeping them on the drawn horizontal line. The germination paper was carefully rolled vertically from the drawn line avoiding seed movement into a moderately tight tube and secured with duct tape. All the tubes were labelled and kept in a warm place at ~ 25 °C for four days before taking the root length readings in mm from seed base to the end of the longest root. An unpaired *t-*test was run to determine if the differences were statistically different. Similar method was followed using the 95 individual Hv584/*rpr9* RILs, Q21861, Hv584 and the *rpr9* mutant and root length was recorded for 3 germinated seeds and data was used for QTL mapping.

### Genotyping and genetic map construction

The Hv584/*rpr9* RIL population was genotyped using the 9 k Illumina Infinium iSelect assay [71] at the USDA cereal genotyping lab, Fargo ND. Markers containing greater than 30% missing data were removed from the data set. Linkage map for the Hv584/*rpr9* RIL population generated using MapDisto 2.1.1 [72]. The command ‘Find linkage groups’ was used to make markers linkage group with a logarithm of the odds (LOD) value of 3.0 and rmax of 0.3. A minimum cutoff score of 10% was used to filter loci with missing data in 2-point matrices. The ‘AutoOrder’, ‘AutoCheckInversions’, and ‘AutoRipple’ commands were utilized to generate the linkage map at a LOD of 3.0 and Kosambi mapping function was used to calculate the genetic distances. The final linkage maps were drawn using ‘Draw all sequences’ command. Co-segregating markers were identified and removed, leaving a single marker within each block of co-segregating loci with the least amount of missing data at each position. For stem rust and seedling root length QTL analysis, the whole map was regenerated without using *rpr9* phenotype as a marker. Non-redundant loci and their corresponding de novo genetic positions and trait data was exported to utilized in QGene 4.4 [73].

### Genetic map concordance

Genetic positions based on POPSEQ [74] were also obtained for each iSelect marker using the POPSEQ position data sheet for iSelect markers downloaded from the Barlex web server (barlex.barleysequence.org). iSelect marker names for the 2701 polymorphic markers for the *rpr9* mapping was imported in the Microsoft Excel and ‘vlookup’ function was used to identify POPSEQ positions. If no POPSEQ position was assigned, then IPK barley BLAST server (https://webblast.ipk-gatersleben.de/barley_ibsc/viroblast.php) based sequence and position search was conducted. Further, Barleymap online tool [75] was used to cross check the iSelect marker anchoring in the POPSEQ positions in the seven barley chromosome.

### Quantitative trait loci analysis

For QTL analysis de novo loci position for non-cosegregating loci was used. The average of three replication of seedling root length data and stem rust phenotyping trait data was assigned for analysis. QTL analysis was conducted using the single trait multiple interval mapping (MIM) algorithm at scanning interval of 5. A permutation test consisting of 1000 iterations was carried out to determine a LOD threshold at the *p* ≤ 0.05 significance level.

## Statistical analysis

Mean Score of categorical disease ITs (0–4) was calculated in MS-Excel-2018. The χ^2^ test (goodness of fit) to determine 3:1 segregation ratio was carried out in SAS 9.4. To determine the root length phenotype, root length was measured in a continuous scale and unpaired t-test was carried out in SAS 9.4 (*p* ≤ 0.05).

### DNA extraction, fragmentation optimization, exome capture library preparation, and sequencing

Eleven seed of wt Q21861, and the *rpr9* mutant were placed on water-soaked Whatman filter paper in a disposable petri dish for 24 h. Embryos were excised using a sterilized DNase free scalpel and a total of five excised embryos were used for DNA extraction using the PowerPlant Pro DNA isolation kit (MoBIO Laboratories Inc., QIAGEN Carlsbad CA). Mechanical lysis of samples was done using a mechanical bead beater at 2000 rpm for 2 cycles of 3 min each and the manufacturer’s protocol was followed for DNA isolation. The quality of extracted DNA was checked by running an aliquot of 1 μL of gDNA on a 1% agarose gel supplemented with GelRED (Biotium) fluorescent nucleic acid dye. The DNA was determined to have good integrity when it showed a high molecular weight band ~ 15–20 kb with minimal low molecular weight smearing indicative of DNA degradation. The gDNA was quantified using the Qubit Broad Range DNA Quantification kit (Thermo Scientific). Enzymatic DNA shearing was optimized to generate desired fragment sizes of 250–450 bp by conducting a time course experiment with digestion reactions consisting of 1.5 μg of gDNA in a 20 μl reaction with NEB dsDNA Fragmentase enzyme, 1x Fragmentase reaction buffer and 10 mM MgCl_2_ (New England Biolabs, Ipswich MA). Digestion reactions were incubated at 37 °C for 10, 15, 20, 25 and 30 min and then inactivated by adding 5 μl of 0.5 M EDTA, followed by AMPure XP magnetic bead DNA purification (Agencourt). DNA size distribution was analyzed on the Agilent 2100 Bioanalyzer (Agilent Technologies) using a DNA 1000 kit (Agilent Technologies) following the manufacturer protocol for chip loading and data analysis. The 25-min enzymatic digestion was found to produce the optimal fragment size distribution ranging between 250 and 450 base pairs and was used to produce fragmented DNA libraries of Q21861, and *rpr9* (Additional file 10: Figure S6).

Fragmented gDNA samples were used for whole exome capture using the Roche NimbleGen SeqCap EZ Developer probe pool barley exome design 120426_Barley_BEC_D04 with a total capture design size of 88.6 Mb. After exome capture the KAPA HTP gDNA library preparation kit was used for Illumina sequencing library preparation. The standard manufacturer protocol was followed for library preparation using the KAPA HTP kit, except for size selection being performed on a Pippin Prep gel purification system (Sage Science) with a 250–450 bp targeted size selection. The gDNA used to prepare the barcoded barley whole exome capture multiplexed library was developed according to seqCAP EZ Library SR user guide 4.1 protocol. Quality and size distribution of the final capture library was determined using a bioanalyzer as previously described. A Qubit fluorometer was used to quantify the library for final dilution and sequencing on an Illumina NextSeq flow cell generating 150 base pair single end reads. Qubit readings were used since the bioanalyzer tends to underestimate the quantity for size selected libraries due to presence of DNA fragments not falling within the desired range (150–450). Thus, posing a possible chance of over-flooding of the flow cells due to the under-diluted library.

### Exome capture data analysis3

The *rpr9* mutant and wildtype Q21861 sequencing reads were parsed by their specific barcodes added during library preparation. The quality scores of the raw sequencing reads were determined using the FQC dashboard [76]. The Illumina reads were imported into CLC Genomics Workbench v8.0 in FASTQ format and trimmed for the presence of adapter sequences. Mutant and wildtype reads were aligned to the barley reference genome and the barley reference gene set using the BWA ‘mem’ algorithm [77] with default settings. The alignments were used to identify deleted region utilizing two separate data analysis pipelines: (1) Small deletions (less than 100 bp) were identified using SAMtools ‘mpileup’ with default settings [78], the identified variants were filtered for a minimum read depth of three and a minimum individual genotype quality of 10 using VCFtools [79], 2) As fast neutron mutagenesis may induce large chromosomal deletions, sequencing coverage was calculated from the reads aligned to the reference gene set using SAMtools ‘idxstats’ to identify full gene deletions. To eliminate reads that map to paralogous genes and therefore may give rise to the identification of false negative deletions, only uniquely mapping reads were extracted using SAMtools ‘view’ and used in coverage analysis. Since we mapped the *rpr9* QTL and determined that ~ 610 high confidence genes are present in between the flanking markers, a ratio of captured reads between *rpr9* and Q21861 was calculated with a cutoff score of 0.05 to identify genes which have very low or no reads in the *rpr9* exome capture. Physical positions of fully deleted exome capture targets were obtained from the barley physical map [39], allowing for the identification of candidate genes within the delimited region and characterization of the larger chromosomal deletions typically containing multiple genes generated with the high energy fast neutron irradiation.

## Additional files

Additional file 1: **Table S1.** Seedling QCCJ phenotyping and root length measurement data on Hv584/rpr9 F_2:6_ RIL population. (XLSX 30 kb)

Additional file 2: **Table S2.** List of 2701 redundant loci across the seven chromosomes of barley used for the genetic map construction. (XLSX 73 kb)

Additional file 3: **Figure S1.** Linkage map of Hv584*/rpr9* F_2:6_ recombinant inbred line population spanning the seven barley chromosomes containing 563 polymorphic non-redundant SNP markers, created using MapDisto software. (PDF 158 kb)

Additional file 4: **Figure S2.** QTL map of *rpr9* region on barley chromosome 3H for average diseases ratings on Hv584/*rpr9* RILs inoculated with *Pgt* race QCCJB. X axis represents the all 90 non-redundant loci (representing 390 redundant loci) on the ch. 3H and Y axis represents the LOD score. Map was generated in QGene 4.4 using single trait MIM algorithm. (JPG 817 kb)

Additional file 5: **Figure S3.** QTL mapping showed that the *rpr9* region on barley chromosomes 3 is the only significant QTL detected for average seedling root length using the Hv584/*rpr9* RIL population. X axis represents the non-redundant loci on the barley chromosomes and Y axis represents the LOD score. Map was generated in QGene 4.4 using single trait MIM algorithm. (JPG 372 kb)

Additional file 6: **Table S3.** List of 487 exome capture target genes under the *rpr9* QTL with their physical position and gene orientation on barley ch. 3H. Exome capture reads for Q21861-wt and Q21861-*rpr9* mutant and their coverage ratio for each target is given. (XLSX 41 kb)

Additional file 7: **Figure S4.** Possible orientation of gene cluster in the *rpr9* region on barley chromosome 3H. (A) Current annotation of gene order in *rpr9* region. (B) Proposed gene order orientation in Q21861-*rpr9* mutant and Q21861-wt for gene HORVU3Hr1G074960 based on the exome capture and SNP marker SCRI-RS-27417 read alignment. Read alignment in Q21861-wt (4839 reads) was present across the CDS and 3′ UTR, however, in Q21861-*rpr9* mutant 269 exome capture reads were aligned to 3′ coding and UTR region which includes the physical position of SNP marker SCRI-RS-27417. In figure, red arrows denote the genes present in the *rpr9* region, black arrows represent the *rpr9* flanking genes and asterisk with vertical black bar shows the position of SNP in marker SCRI-RS-27417 on chromosome 3H represented by horizontal white bar. (PDF 1320 kb)

Additional file 8: **Figure S5.** Average root length distribution for Hv584 x *rpr9* F_6_ RIL population (yellow bars) along with wild type Q21861 (green bar), mutant *rpr9* (red bar) and Hv584 (blue bar) in increasing order. The Y-axis represents root length in millimeters and X-axis denote 95 Hv584 x *rpr9* F_6_ RILs with parents used for root length measurement. (JPG 1239 kb)

Additional file 9: **Table S4.** List of fifteen annotated high confidence *SKP-1* like genes in barley whole genome assembly. (XLSX 51 kb)

Additional file 10: **Figure S6.** Size determination of fractionated DNA after 25 min of barley DNA digestion with NEB DNA fragmentize enzyme on Bioanalyzer 1000 DNA chip. A 25-min enzymatic digestion produced DNA fragments in the size range of 250–450 base pairs, which was required for exome capture library preparation. The X-axis represents the fluorescence units and Y-axis represents the size of DNA fragments. The two terminal peaks represent the DNA ladder peaks with the lower marker at 25 base pairs (left) and the upper marker 1000 base pairs (right). (JPG 950 kb)

## Data Availability

All the material and data can be obtained from the authors upon request through the Barley Pathology lab, Department of Plant Pathology at North Dakota State University.

## Abbreviations

ABA: Abscisic acid
bp: Base pair
CDS: Coding determining sequence
ch.: Chromosome
cM: Centi Morgan
CNL: Coiled coil-NB-LRR
ETI: Effector triggered immunity
FN: Fast neutron
HPI: Hours post inoculation
HR: Hypersensitive response
IT: Infection type
LRK: Receptor like kinase
Mbp: Million base pair
*NbSGT1*: *Nicotiana benthamiana* suppressor of G2 allele of SKP1
NLR: Nucleotide-binding domain and leucine-rich repeat
NQO: NAD(P)H-quinone oxidoreductase
PAMP: Pathogen associated molecular pattern
PCD: Programmed cell death
*Pgt*: *Puccinia graminis* f. sp. *tritici*
PR: Pathogen related
PRRs: Pattern recognition receptors
PTI: PAMP triggered immunity
QTL: Quantitative trait loci
RIL: Recombinant inbred line
RLKs: Receptor-like kinases
*Rme1*: *rpg4* modifier element 1
RMRL: *rpg4*-mediated resistance locus
RMRL2: *rpg4*-mediated resistance locus 2
*rpr9*: Required for *P. graminis* resistance 9
SCF: SKP/Cullin/F-box and ring finger protein Rbx1
SKP1: S-phase kinase- associated protein
STPK: Serine / threonine protein kinase
TNL: Toll/interlukin 1 receptor-Nucleotide binding-Leucine rich repeats
UTR: Untranslated region
wt: Wild type
