# Supplementary figures and images for: Characterization of genes required for both Rpg1 and rpg4-mediated wheat stem rust resistance in barley
Source: BMC Genomics. 2019 Jun 14;20:495. doi: 10.1186/s12864-019-5858-z (PMC6570958; doi:10.1186/s12864-019-5858-z)

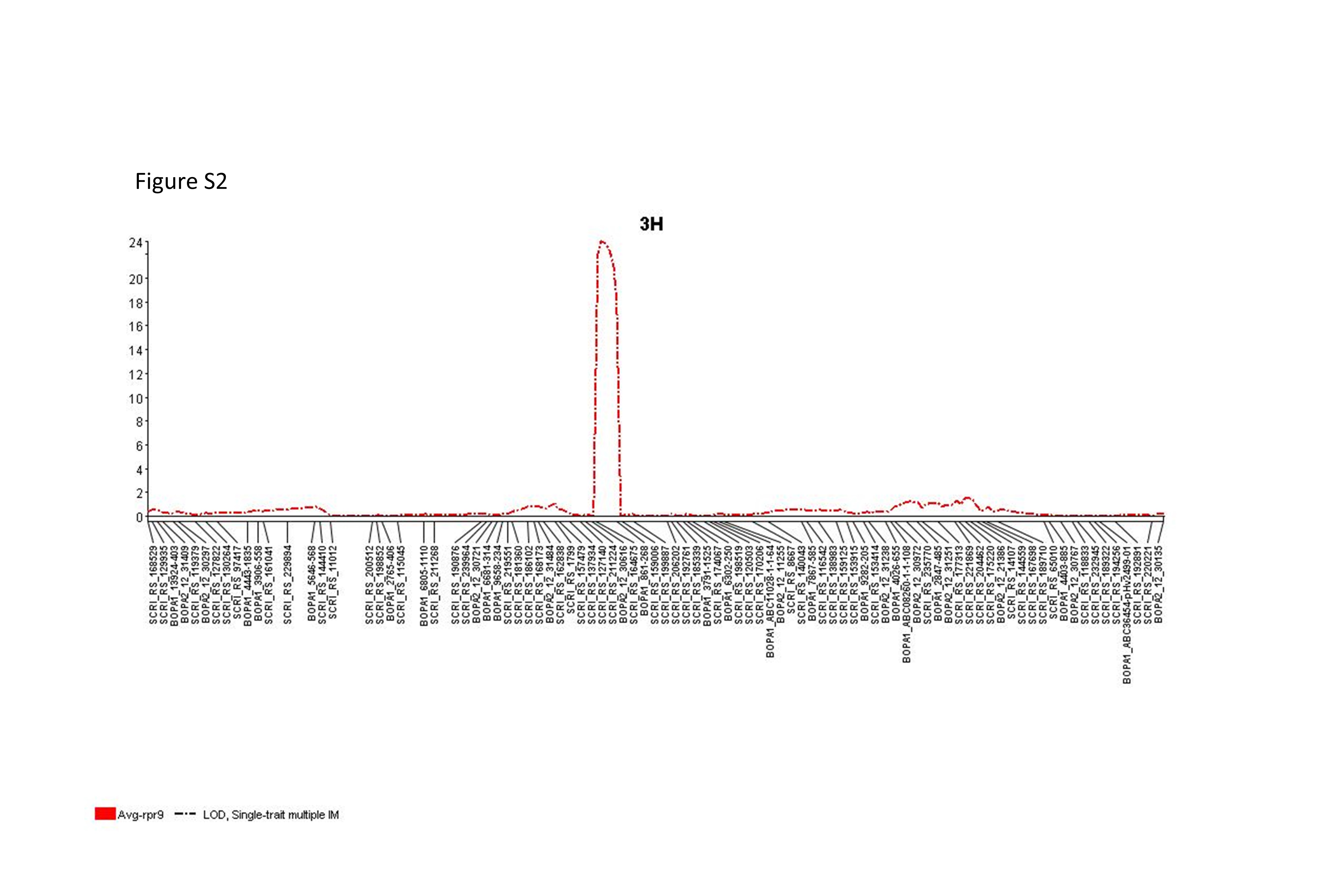

Supplement: Supplementary file 4 — Figure S2. QTL map of rpr9 region on barley chromosome 3H for average diseases ratings on Hv584/rpr9 RILs inoculated with Pgt race QCCJB. X axis represents the all 90 non-redundant loci (representing 390 redundant loci) on the ch. 3H and Y axis represents the LOD score. Map was generated in QGene 4.4 using single trait MIM algorithm. (JPG 817 kb) [file 12864_2019_5858_MOESM4_ESM.jpg]

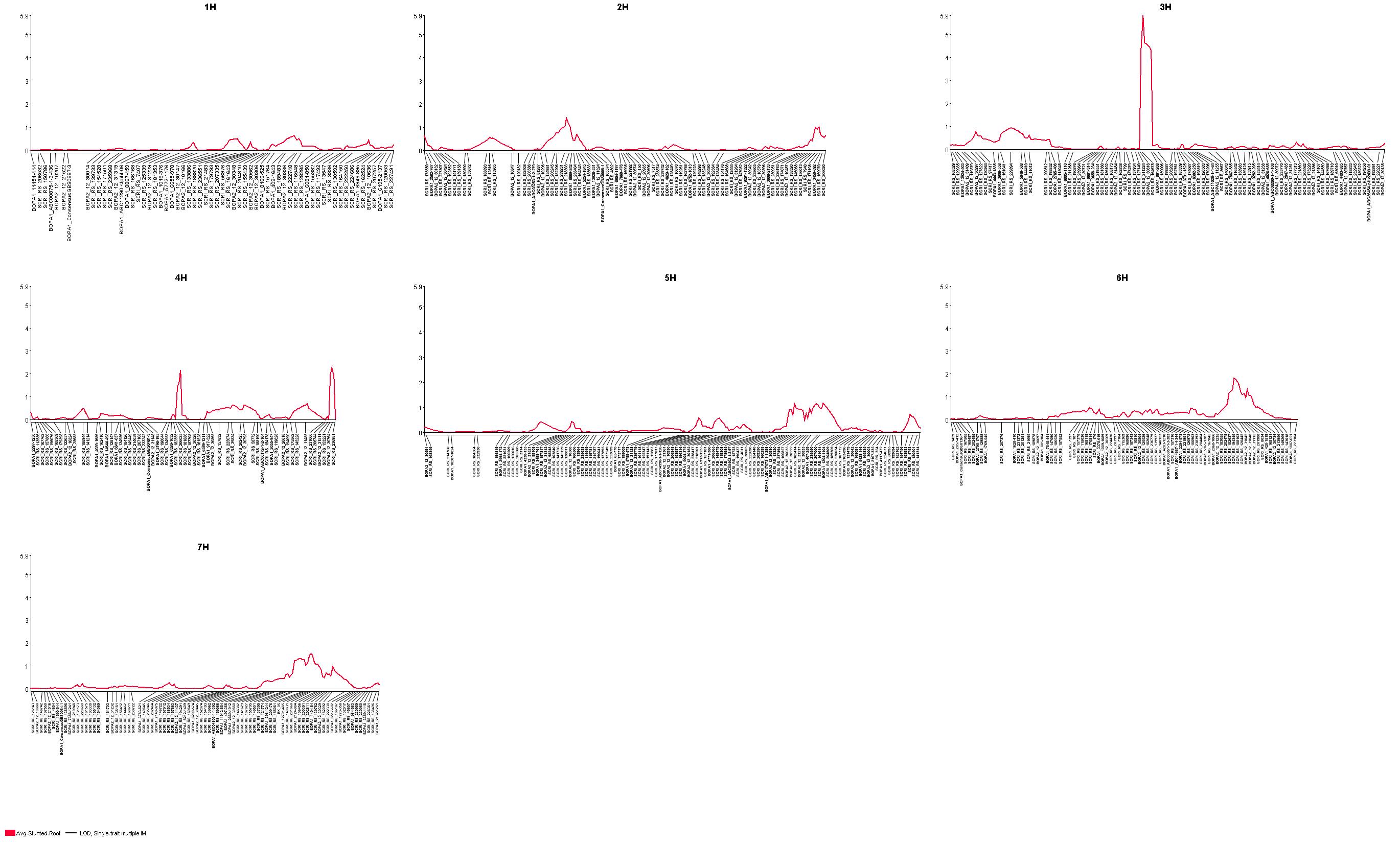

Supplement: Supplementary file 5 — Figure S3. QTL mapping showed that the rpr9 region on barley chromosomes 3 is the only significant QTL detected for average seedling root length using the Hv584/rpr9 RIL population. X axis represents the non-redundant loci on the barley chromosomes and Y axis represents the LOD score. Map was generated in QGene 4.4 using single trait MIM algorithm. (JPG 372 kb) [file 12864_2019_5858_MOESM5_ESM.jpg]

Figure S4

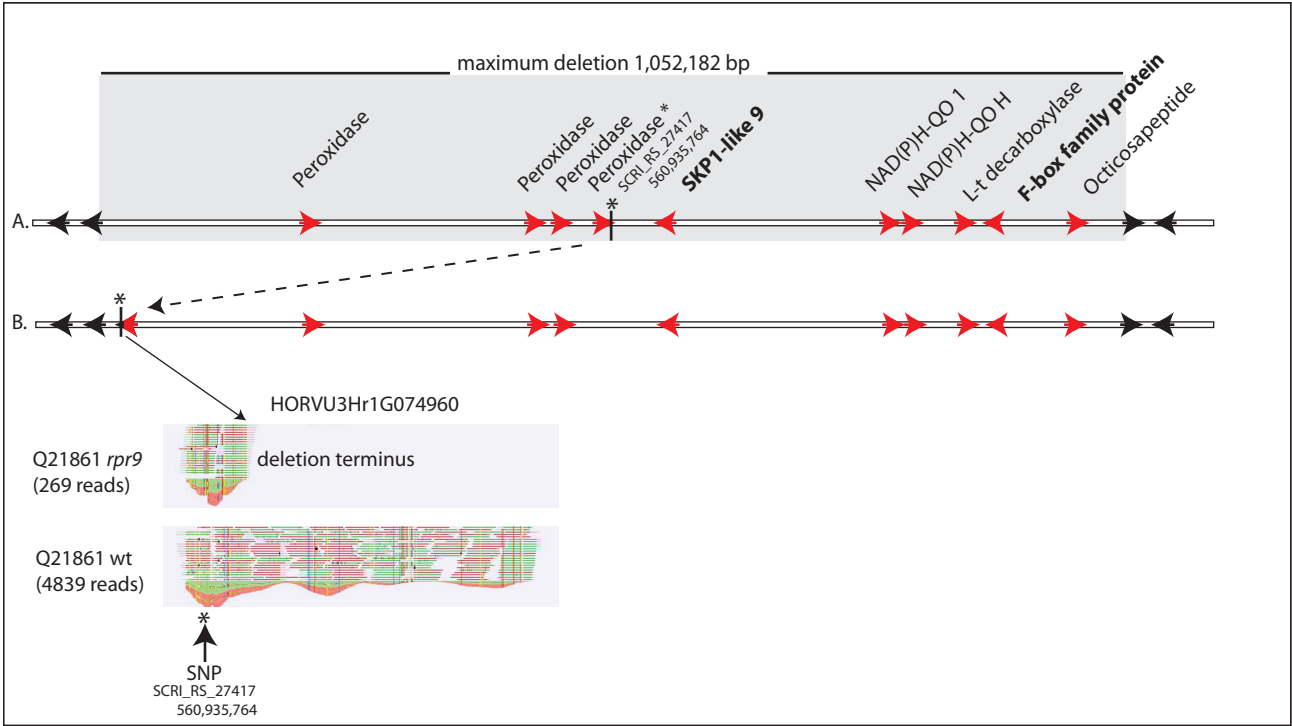

Supplement: Supplementary file 7 — Figure S4. Possible orientation of gene cluster in the rpr9 region on barley chromosome 3H. (A) Current annotation of gene order in rpr9 region. (B) Proposed gene order orientation in Q21861-rpr9 mutant and Q21861-wt for gene HORVU3Hr1G074960 based on the exome capture and SNP marker SCRI-RS-27417 read alignment. Read alignment in Q21861-wt (4839 reads) was present across the CDS and 3′ UTR, however, in Q21861-rpr9 mutant 269 exome capture reads were aligned to 3′ coding and UTR region which includes the physical position of SNP marker SCRI-RS-27417. In figure, red arrows denote the genes present in the rpr9 region, black arrows represent the rpr9 flanking genes and asterisk with vertical black bar shows the position of SNP in marker SCRI-RS-27417 on chromosome 3H represented by horizontal white bar. (PDF 1320 kb) [file 12864_2019_5858_MOESM7_ESM.pdf]

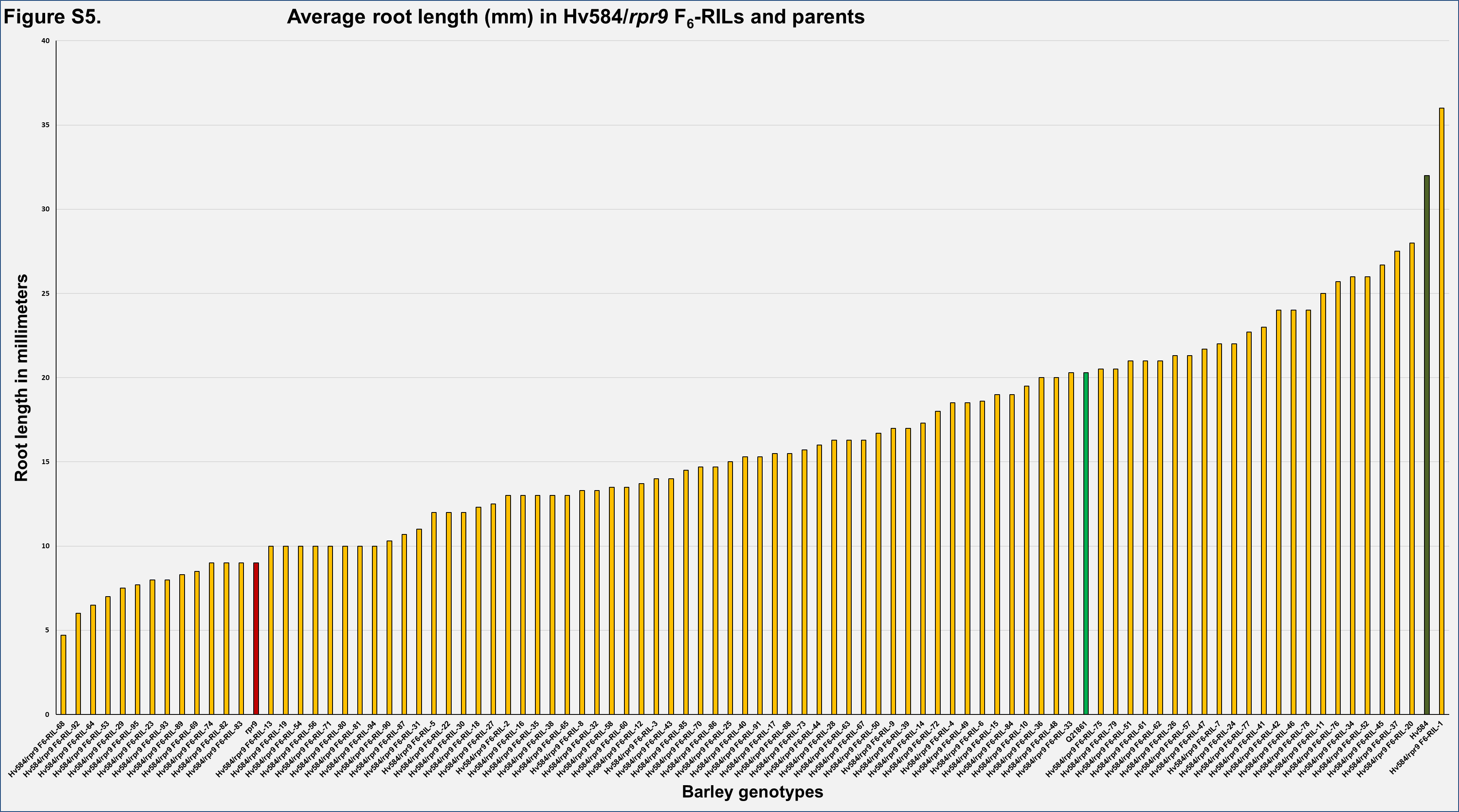

Supplement: Supplementary file 8 — Figure S5. Average root length distribution for Hv584 x rpr9 F6 RIL population (yellow bars) along with wild type Q21861 (green bar), mutant rpr9 (red bar) and Hv584 (blue bar) in increasing order. The Y-axis represents root length in millimeters and X-axis denote 95 Hv584 x rpr9 F6 RILs with parents used for root length measurement. (JPG 1239 kb) [file 12864_2019_5858_MOESM8_ESM.jpg]

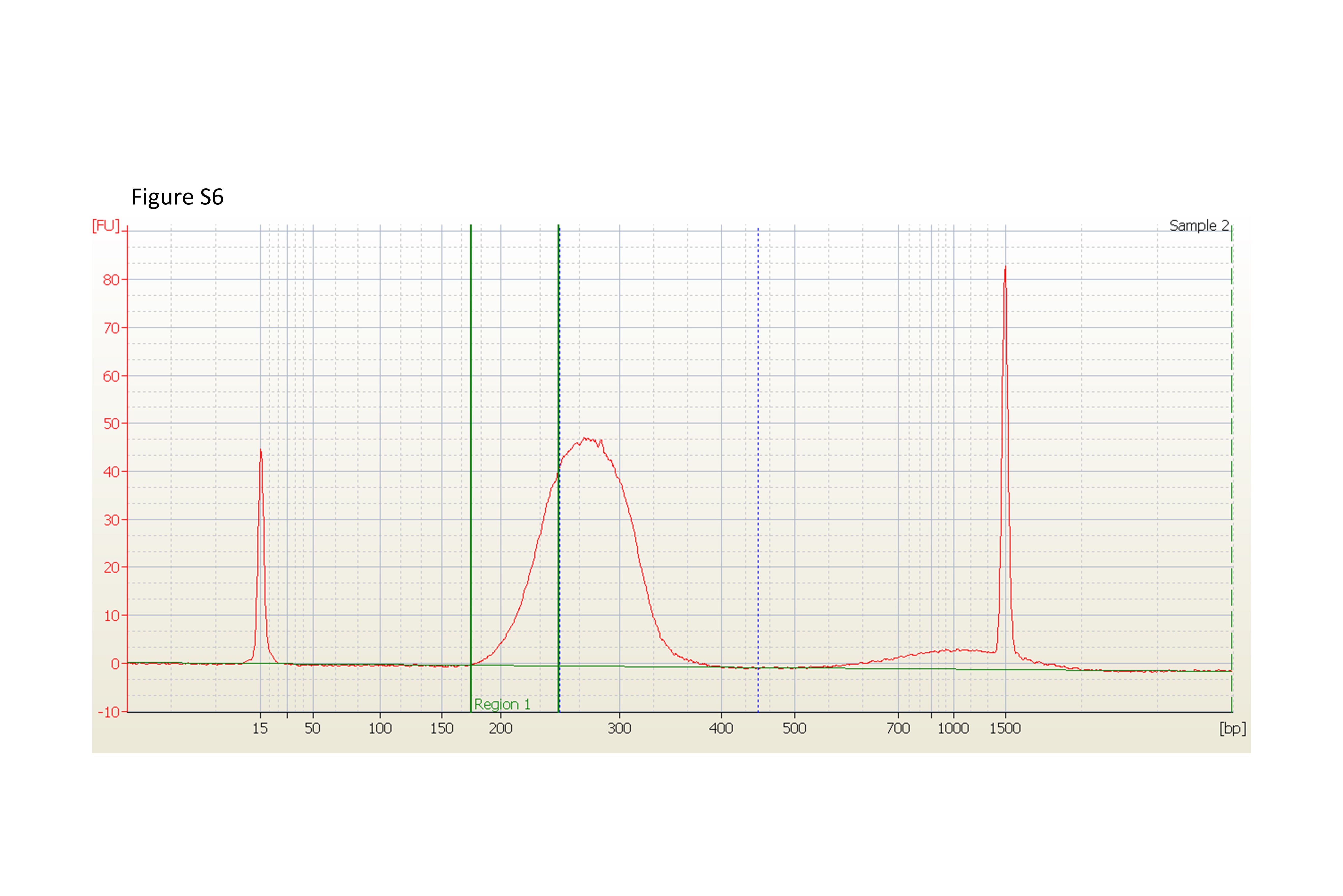

Supplement: Supplementary file 10 — Figure S6. Size determination of fractionated DNA after 25 min of barley DNA digestion with NEB DNA fragmentize enzyme on Bioanalyzer 1000 DNA chip. A 25-min enzymatic digestion produced DNA fragments in the size range of 250–450 base pairs, which was required for exome capture library preparation. The X-axis represents the fluorescence units and Y-axis represents the size of DNA fragments. The two terminal peaks represent the DNA ladder peaks with the lower marker at 25 base pairs (left) and the upper marker 1000 base pairs (right). (JPG 950 kb) [file 12864_2019_5858_MOESM10_ESM.jpg]
