# Supplementary material for: Characterization of genes required for both Rpg1 and rpg4-mediated wheat stem rust resistance in barley
Source: BMC Genomics. 2019 Jun 14;20:495. doi: 10.1186/s12864-019-5858-z (PMC6570958; doi:10.1186/s12864-019-5858-z)
Supplement: Supplementary file 3 — Figure S1. Linkage map of Hv584/rpr9 F2:6 recombinant inbred line population spanning the seven barley chromosomes containing 563 polymorphic non-redundant SNP markers, created using MapDisto software. (PDF 158 kb) [file 12864_2019_5858_MOESM3_ESM.pdf]

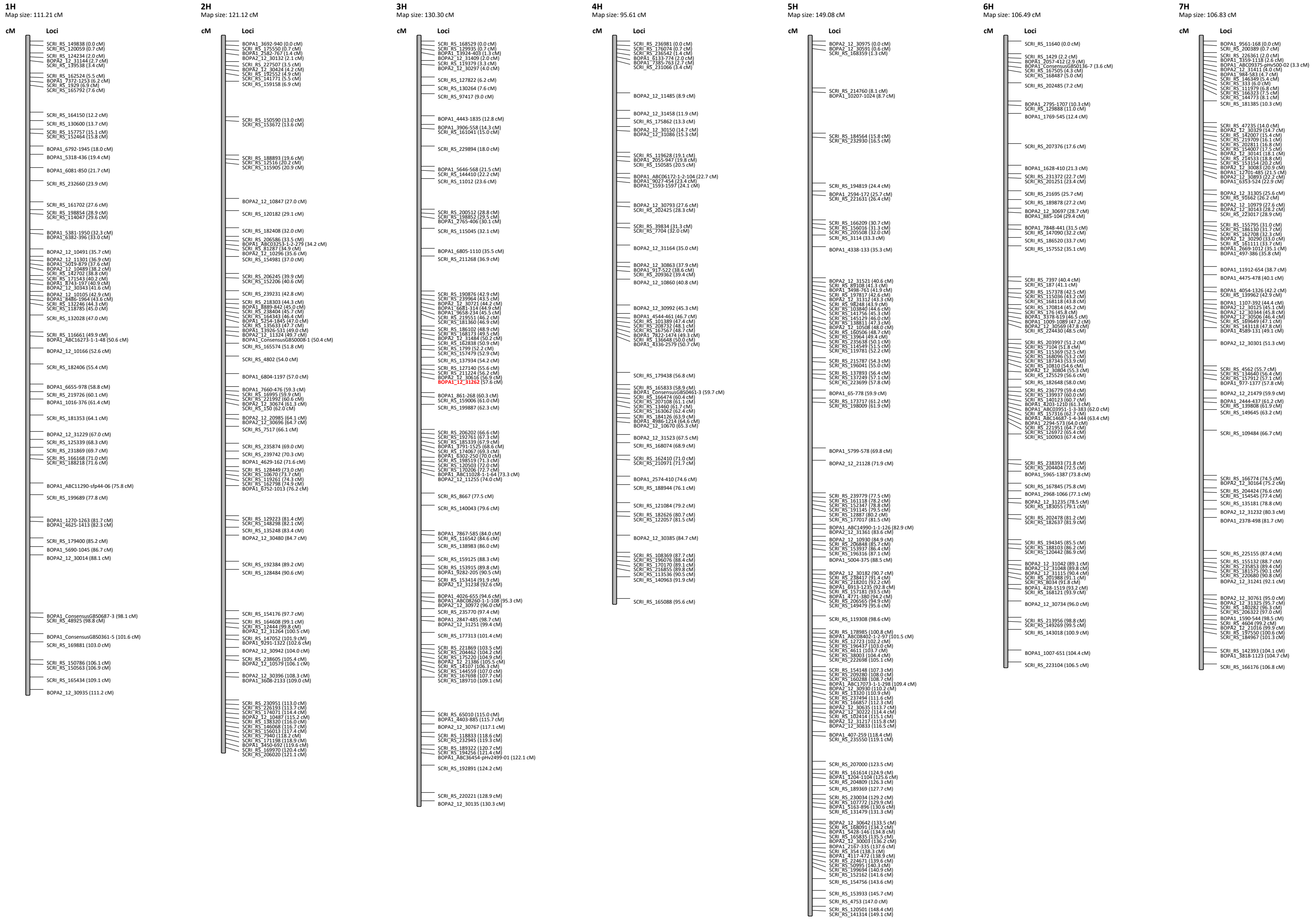

1H
Map size: 111.21 cM
2H
Map size: 121.12 cM
3H
Map size: 130.30 cM
4H
Map size: 95.61 cM
5H
Map size: 149.08 cM
6H
Map size: 106.49 cM
7H
Map size: 106.83 cM
cM
Loci
